# Keratin 7 expression in different anatomical parts of colonic epithelium in inflammatory bowel diseases and its prognostic value: a 3-year follow-up study

**DOI:** 10.1038/s41598-023-39066-w

**Published:** 2023-07-24

**Authors:** Mervi Tenhami, Lauri Polari, Harry Kujari, Eliisa Löyttyniemi, Diana M. Toivola, Markku Voutilainen

**Affiliations:** 1 Division of Digestive Surgery, Turku University Hospital and University of Turku, P.O. Box 52, 20521 Turku, Finland; 2 Cell Biology, Biosciences, Åbo Akademi University, Turku, Finland; 3 InFlames Research Flagship Center, Åbo Akademi University, Turku, Finland; 4 Department of Pathology, University of Turku and Turku University Hospital, Turku, Finland; 5 Department of Biostatistics, University of Turku and Turku University Hospital, Turku, Finland; 6 Department of Medicine, University of Turku and Turku University Hospital, Turku, Finland

**Keywords:** Biomarkers, Gastroenterology, Pathogenesis

## Abstract

The diagnosis of inflammatory bowel diseases (IBD) may be challenging and their clinical course, characterized by relapses and spontaneous or drug-induced remissions, is difficult to predict. Novel prognostic biomarkers are needed. Keratin 7 (K7) is a cytoskeletal intermediate filament protein which is not normally expressed in the colonic epithelium. It was recently shown that K7 expression in the colonic epithelium is associated with ulcerative colitis and Crohn’s disease, the two main subtypes of IBD. Here we investigated IBD associated K7 neo-expression in different regions of colon and terminal ileum. The correlation of the K7 expression with the inflammatory activity of the epithelium was analyzed in each region. The prognostic value of K7 was estimated by comparing the clinical disease activity after 3 years with the K7 expression at the time of enrollment. Our data shows that the level of K7 expression in inflamed epithelium varies depending on the anatomical region and it is the most pronounced in ascending and descending colon, but it did not predict the severity of IBD for the following 3 years. These results warrant future studies focusing on the biological role of K7 in colon and its utilization as potential IBD biomarker.

## Introduction

Inflammatory bowel disease (IBD) is a general term for chronic intestinal inflammation, and it usually refers to ulcerative colitis (UC) and Crohn’s disease (CD). The clinical course of IBD is characterized by relapses and spontaneous or drug-induced remissions. The etiology of the disease is unclear, but the mechanisms involve genetic susceptibility, predisposive environmental factors and dysbiosis of intestinal bacteria^1^. At present, IBD is a global disease, the prevalence being highest (ca 0.3%) in developed countries. The incidence increase of both UC and CD has stabilized in the US and Europe, but it is accelerating in newly industrialized countries where the annual percentage increase is 5–18% for CD and 10–20% for UC^2^.

The diagnosis of IBD is based on endoscopic examination and mucosal biopsies with clinical symptoms, laboratory tests and imaging studies^1^. Still, the course of disease cannot be predicted by currently known parameters. The only prognostic attribute is the previous disease activity^3^. A predictive marker would be beneficial in aiding decision making when choosing proper medication with the least adverse effects^4^, and possibly by indicating risk for severe form of the disease or even the risk for colorectal cancer.

Keratins (K) are ubiquitous intracellular intermediate filament proteins expressed mainly in epithelial cells. They are major components of the cytoskeleton and they have been shown to protect cells from mechanical and non-mechanical stress. They also regulate electrolyte transport in colonocytes, participate in cellular differentiation and proliferation, and they may have a role in inflammatory signaling^5–9^. The most common simple intraepithelial keratins are K7, K8, K18, K19 and K20, and they are divided into acidic I (K18–K20) and basic II (K7 and K8) type keratins. Type I and II keratins form non-covalent obligate heteropolymers in a 1:1 ratio, and they are expressed in a tissue-specific manner^10^. In the intestinal epithelium, the principally expressed keratins are K8, K18, K19 and K20^9^. K20 is expressed in luminal part of crypts in intestinal mucosa, and as it is mostly colon specific, it is used as a marker for metastases originating from colorectal carcinoma^11^ with staining for CDX-2 and SATB2. K7 forms part of the cytoskeleton in glandular and ductal epithelial cells in ovary, endometrium, breast, lung, urinary bladder, and bile ducts, but it is not expressed in healthy colonic epithelium^12–17^. Its disease-expression patterns and molecular roles are not well known as compared to other simple epithelial keratins^18^.

The colonic epithelial barrier is damaged in IBD accompanied by erosion, edema, crypt distortion and branching, and followed by regeneration^19,20^. Fecal calprotectin reflects the neutrophilic activity in intestinal tract but currently there is no clinically usable molecular marker that would indicate disruptions in epithelial integrity or that would predict the clinical course of the disease^19^. Among the homeostasis maintaining epithelial components keratins are crucial cytoskeletal proteins and their expression is changed in colonic stress conditions^21^. Further, keratins have been shown to react to stress in other tissues and upregulation of several keratin genes are associated with cancer progression^22^. K7 is found to be expressed in UC-related neoplasms^23,24^, and we previously found that K7, not expressed in healthy colonic epithelium, is significantly upregulated in IBD and the highest focal K7 positive cell levels were found to be associated with the most severe epithelial damage and the samples with granulomas close to epithelium had increased number of K7 positive cells^17^.

Based on the different embryological origins, colon can be divided anatomically into a proximal part including cecum, ascending and transverse colon, and a distal part that covers descending and sigmoid colon as well as rectum. The main functions, like water and ion absorption, and protection against bacteria, are considered similar throughout the colon, but regional variation exists as suggested by development of UC from the rectum towards the proximal colon. It is also typical that CD affects discontinuous segments of the intestinal epithelium. Proteomic studies have shown that there is variation in expression of several transporter proteins in different parts of colon^25–27^, and the amount of proteins involved with bacterial sensing decrease towards distal colon, while the amount of proteins associated with microbial defense increase towards distal colon^28^. In addition, Boyer et al. have reported that in physiological condition in colon the total amount of immune cells decreases from cecum to rectum^29^. In IBD, the expression of K7 in various parts of colon and ileum is unknown.

The over-expression of K7 is associated with poor prognosis in several adenocarcinomas^30–32^, and a recent study has suggested that artificial intelligence based analysis of K7-stained liver specimen can predict disease outcome in primary sclerosing cholangitis^33^. The role of K7 in the prognosis of IBD is not known.

In this study we aimed to explore further the association between K7 expression and intestinal inflammation caused by IBD in distinct segments of colon, and we also included terminal ileum which is the most often affected part of the intestine in CD. The prognostic value of K7 for the severity of IBD was also estimated by a 3 year-follow up study.

## Methods

Sixteen patients with known or suspected IBD were recruited into this study. The recruitment protocol and exclusion criteria are described in the previous study^34^. The research grant of Turku university hospital was received by the director of the Division of medicine (decision TO5/039/14; 13604). The patients were informed about the nature of the study, and they signed an informed consent with the protocol approved by the ethics committee of the Hospital District of Southwest Finland (T31/2014) before participation. The study was conducted in accordance with the Declaration of Helsinki and all the methods were performed in accordance with the relevant guidelines and regulations.

Intestinal inflammation was assessed with ileocolonoscopy and routine biopsies were taken from terminal ileum, ascending colon, transverse colon, descending colon, and rectum. HE-staining and K7-immunostaining were performed according to the routine laboratory protocols of the Department of Pathology of Turku University Hospital, Finland, and they are described elsewhere^17^. K20-immunostaining was performed according to the standard protocol of the Department of Pathology of Turku University Hospital, the monoclonal primary anti (cyto-)keratin clone used was SP33 (Roche/Ventana) for IVD use. The quality organization of the pathology laboratory is audited according to the specifications of the international standard SFS-EN ISO 15189 by FINAS (Finnish accreditation services), and all staining procedures are validated per antibody lot before use. Internal positive control tissue is included in all slides in routine use. The inflammatory activity in each biopsy was graded into four classes by a pathologist specialized in gastrointestinal pathology: (1) no activity; (2) mild (cryptitis); (3) moderate (crypt abscesses); and (4) severe (erosions/ulcers) according to ECCO guidelines^35^. The bioimage analysis based on optical density (OD) of K7 and K20 stainings was performed using QuPath software version 0.2.3 run on Windows^36^. The percentage of the K7 positive cells in each histological sample was calculated by manually annotating the region of interest (ROI) including one thousand epithelial cells per biopsy. The ROI was chosen so that it represents the general K7 expression level of the whole histological sample. Whole crypts were preferred to those with discontinuous sites. The immunostaining intensity was used to grade each cell into one of the following classes: (0) no K7 expression; (1) low K7 expression; (2) moderate K7 expression; and (3) high K7 expression by using the QuPath positive cell detection tool (immunostaining cell mean OD). The lowest K7 threshold was based on barely visible cytoplasmic staining^17^. The epithelial cells graded from low to high K7 expression are referred as K7 positive. Fecal calprotectin was measured and Clinical Colitis Activity Index (CCAI)^37^ or Crohn’s Disease Activity Index (CDAI)^38,39^ were calculated for each patient.

The 3-year-follow up study was conducted by collecting data in medical reports in hospital records. The severity of the disease of each patient was graded by an experienced gastroenterologist either to (1) mild/moderate, or (2) severe form based on the need for immunomodulatory or biological therapy, number of relapses per year, need for hospitalization and complications caused by the disease (e.g., stricture formation in CD) during the follow-up time.

### Statistical analysis

**Figure 1:**
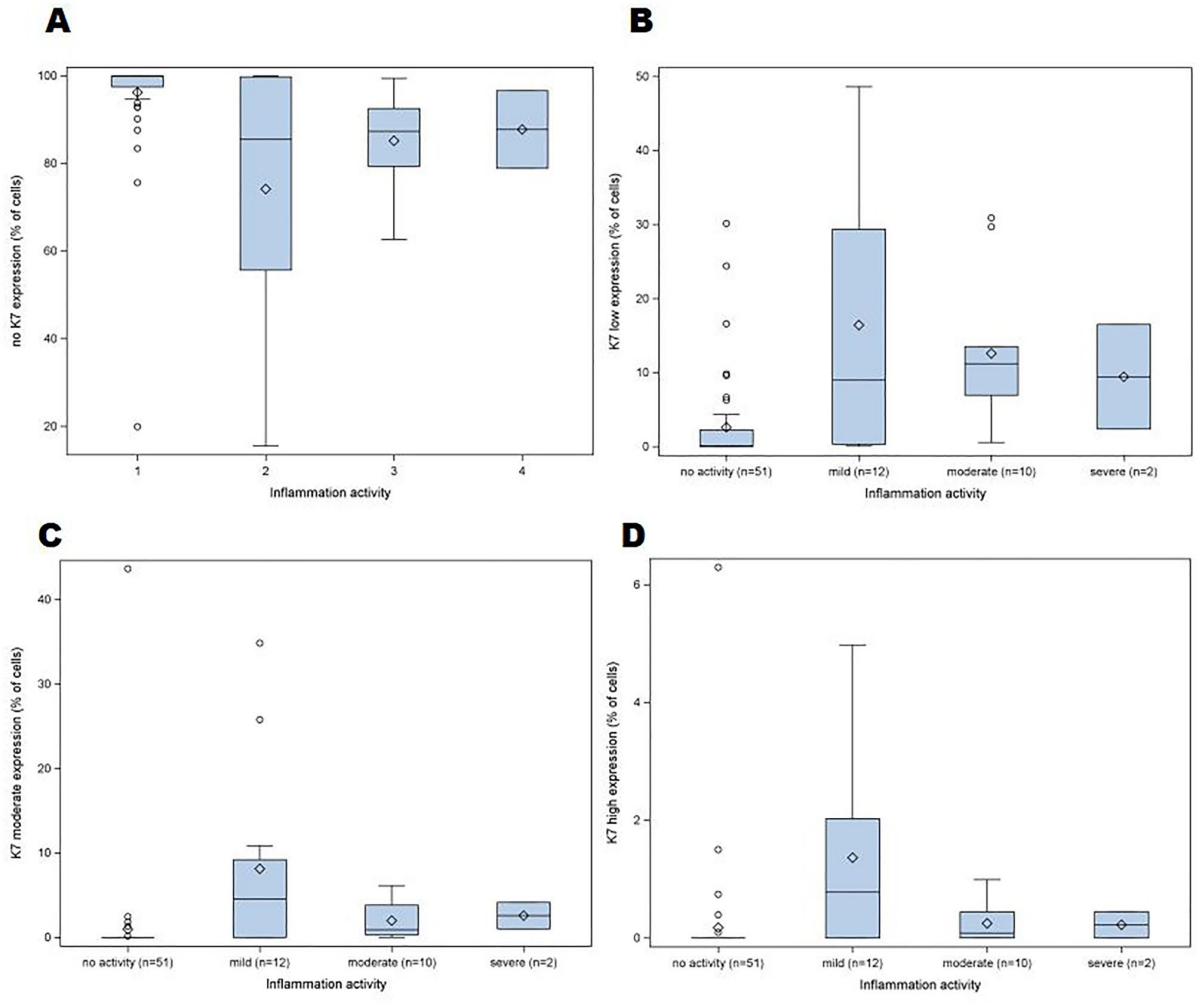
The distribution of the percentage of epithelial cells that express K7 in different grades of inflammation. The percentage of the epithelial cells with no K7 expression (**A**); low K7 expression (**B**); moderate K7 expression (**C**) and high K7 expression (**D**) in biopsies with different grades of inflammatory activity: (1) no activity—(2) mild inflammation—(3) moderate inflammation—(4) severe inflammation. The upper and the lower quartiles are shown by whiskers, the diamond symbol represents the mean, and the circles represent single biopsies.

The relationship between the percentage of K7-positive cells and inflammatory activity was analyzed with one-way analysis of variance separately for each region of biopsy. For K7-positive cells square root transformation was used to fulfill assumption of normally distributed residuals. To study the association of the severity of illness (mild/moderate and severe) and the expression of K7 at the time of enrollment, the K7 expression in ascending colon and descending colon, and in the biopsy site with the greatest K7 expression was evaluated using log-binomial model, including one K7 feature at a time. To illustrate the percentage of epithelial cells that express K7 in different grades of inflammation, four boxplots were drawn (Fig. 1). The Spearman correlation coefficient was calculated to study association between K7 and K20 (DAB) intensities and figure with linear regression line illustrates the phenomenon. The data analysis for this study was done using SAS software, Version 9.4 of the SAS System for Windows (SAS Institute Inc., Cary, NC, USA).

## Results

The demographics of the patients, the fecal calprotectin measured at the beginning of the symptoms, the endoscopic diagnosis, and the medical treatment at the time of enrollment, and the severity of the disease during a 3-year follow-up period are presented in Table 1.

**Table 1:** Patient demographics.

Sex	Age (years)	F-calpro (μg/g)	UC/CD	Endoscopic diagnosis	Medication	Severity of the disease during the 3-year follow-up
F	39	> 2000	UC	proctitis	prednisolone + azathioprine 150 mg	severe
F	19	1400	UC	pancolitis	methylprednisolone + azathioprine 25 mg × 2 + 5-ASA 1600 mg × 2	severe
F	28	> 2000	UC	pancolitis	prednisolone + 5-ASA 1600 mg × 2	mild/moderate
M	27	> 2000	CD	jejunitis, ileitis	methylprednisolone	severe
F	19	> 2000	UC	pancolitis	prednisolone + 5-ASA 1600 mg × 2	severe
F	19	> 2000	I	pancolitis	methylprednisolone + 5-ASA 2 g + 1 g	severe
F	31	> 2000	UC	pancolitis	methylprednisolone + azathioprine 150 mg + 5-ASA 1600 mg × 2	severe
F	30	1294	CD	ileitis, sigmoiditis	methylprednisolone + azathioprine 25 mg × 2	mild/moderate
F	36	393	CD	ileitis	prednisolone + mercaptopurine 50 mg × 1	mild/moderate
F	54	557	CD	ileitis	budesonide 9 mg × 1 + azathioprine 25 mg × 1	mild/moderate
M	37	5860	UC	pancolitis	prednisolone + azathioprine 25 mg × 2	severe
F	19	3178	CD	ileitis, segmental colitis	methylprednisolone	mild/moderate
F	85	45	CD	Segmental colitis	budesonide 9 mg × 1	mild/moderate
M	79	607	CD	jejunitis, ileitis	budesonide 9 mg × 1	mild/moderate

F, Female, M, Male, F-calpro, Fecal calprotectin, UC, Ulcerative colitis, CD, Crohn’s disease, I, Intermediate colitis. The medication is described at beginning the of the diagnosed relapse. The dose of intravenous methylprednisolone was 40 + 20 mg per day (for the hospitalized patients), and the dose of per oral prednisolone (for the patients at outpatient clinic) was 40 mg per day and the cut down of prednisolone was − 5 mg per week consecutively. The dose of oral budesonide was 9 mg per day at the time of relapse and the cut down of the dose was − 3 mg per month consecutively. 5-ASA = 5-aminosalicylic acid. All the parameters besides the severity of the disease are described at the time of enrollment. The severity of the disease is described during the 3-year follow up.

In total, 67 biopsies of bowel were included in the analysis. The lowest percentage of K7-positive epithelial cells was 0.0 in bowel segments with no inflammation and the highest percentage of K7-positive epithelial cells was 85 in a bowel segment with mild inflammation in UC. The K7 expression in terminal ileum was very low and the highest percentage of K7 positive cells was 0.4 in mild ileitis. In inflamed colonic epithelium, the median percentage of low K7 expressing cells was 9–11 of all the epithelial cells. The percentages of moderate and high K7 expressing cells were 1–5 and 0–1, respectively. There was no statistically significant difference between K7 expression in mild, moderate, and severe inflammation (Fig. 1).

**Figure 2:**
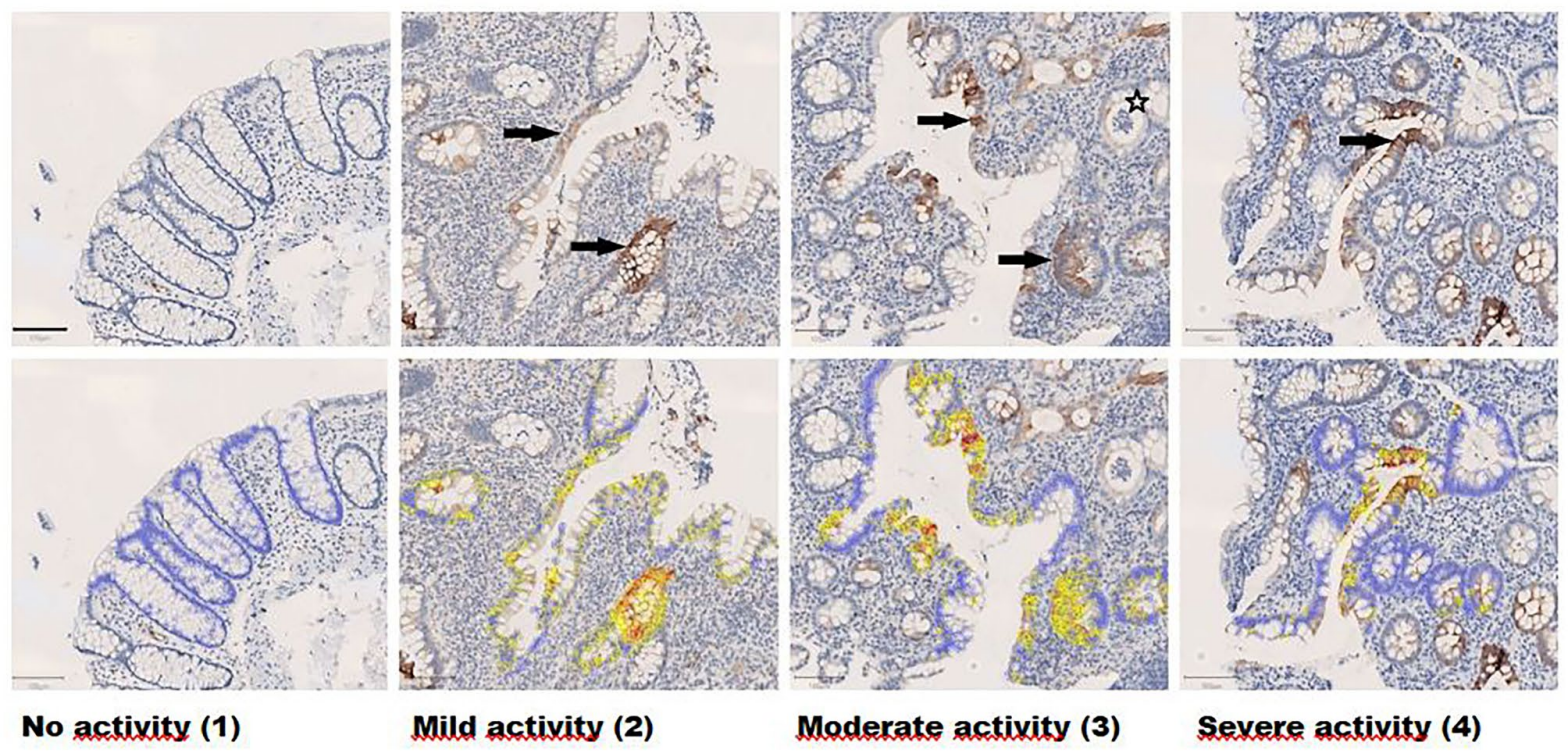
K7-positive cells (brown) in intestinal epithelium in areas representing different grades of inflammatory activity. The arrows point towards K7-positive cells, a crypt abscess is pointed out by a star. The scale bar in the lower left corner equals 100 μm. The lower row from left to right also shows annotated epithelial cells: blue annotation: no K7 expression, yellow annotation: low K7 expression, orange annotation: moderate K7 expression, and red annotation: high K7 expression.

**Figure 3:**
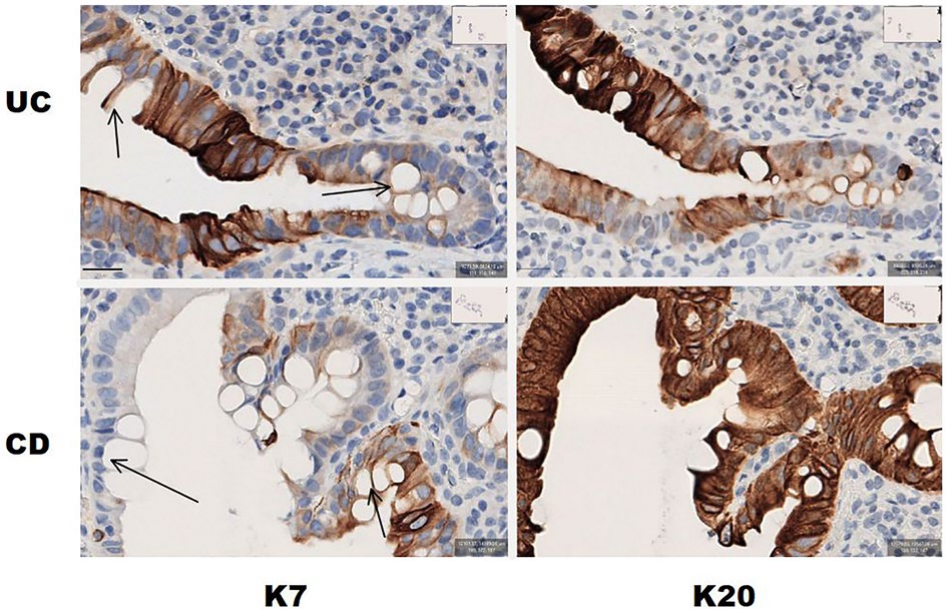
K7 and K20 expression in colonocytes in UC and CD. Higher magnification images of K7 and K20 IHC staining of the biopsy of UC patient and CD patient. The arrows in K7 IHC-stained images point towards goblet cells. The scalebar down left equals 20 μm.

Representative histological sections of K7-immunostained biopsies of patients with no active inflammation–mild inflammation–moderate inflammation and severe inflammation are shown in Fig. 2. The cellular distribution of K7 in colonocytes is seemingly similar to other intestinal keratins such as K8 and K19^40^, also witnessed here in K7-positive colon carcinoma cells (Supplementary Fig. S1). IBD-related K7-positivity was not associated to specific cell subtypes, since both K7 positive and negative colonocytes and goblet cells were found in the same areas in biopsies (Fig. 3).

The relationship between the percentage of the K7-positive epithelial cells and inflammatory activity was studied with one-way analysis of variance separately for each region of biopsy which showed that the inflammatory activity was associated with statistically significant increase in K7-expression in the ascending (*p* = 0.004) and descending colon (*p* = 0.0004), but inflammation did not affect K7-expression in ileum, transverse colon, or rectum, in which the K7 expression was generally lower (Table 2).

**Table 2:** The association between inflammation and K7 expression in distinct sites of the lower gut.

Biopsy site	Represented inflammatory activity	Number of biopsies	K7 positive epithelial cells median%	*p*-value
Ileum	1	11	0.1	
	2	4	0.2	0.87
Ascending colon	1	8	0.6	
	2	3	30	0.004*
	3	4	17	
Transverse colon	1	8	1.1	
	2	2	13	0.14
	3	1	8.6	
	4	1	21	
Descending colon	1	8	0.9	
	2	2	63	0.0004*
	3	5	7.4	
Rectum	1	8	0.5	
	2	1	13	0.23
	4	1	3.4	

The inflammatory activity, number of biopsies in each class, median percentage of K7( +) cells in sites of biopsy from ileum to rectum, and statistical significance of the difference (*p*-values) in K7 expression between normal and inflamed epithelial cells in terminal ileum and distinct sites of colon. *p < 0.05 was considered statistically significant.

**Figure 4:**
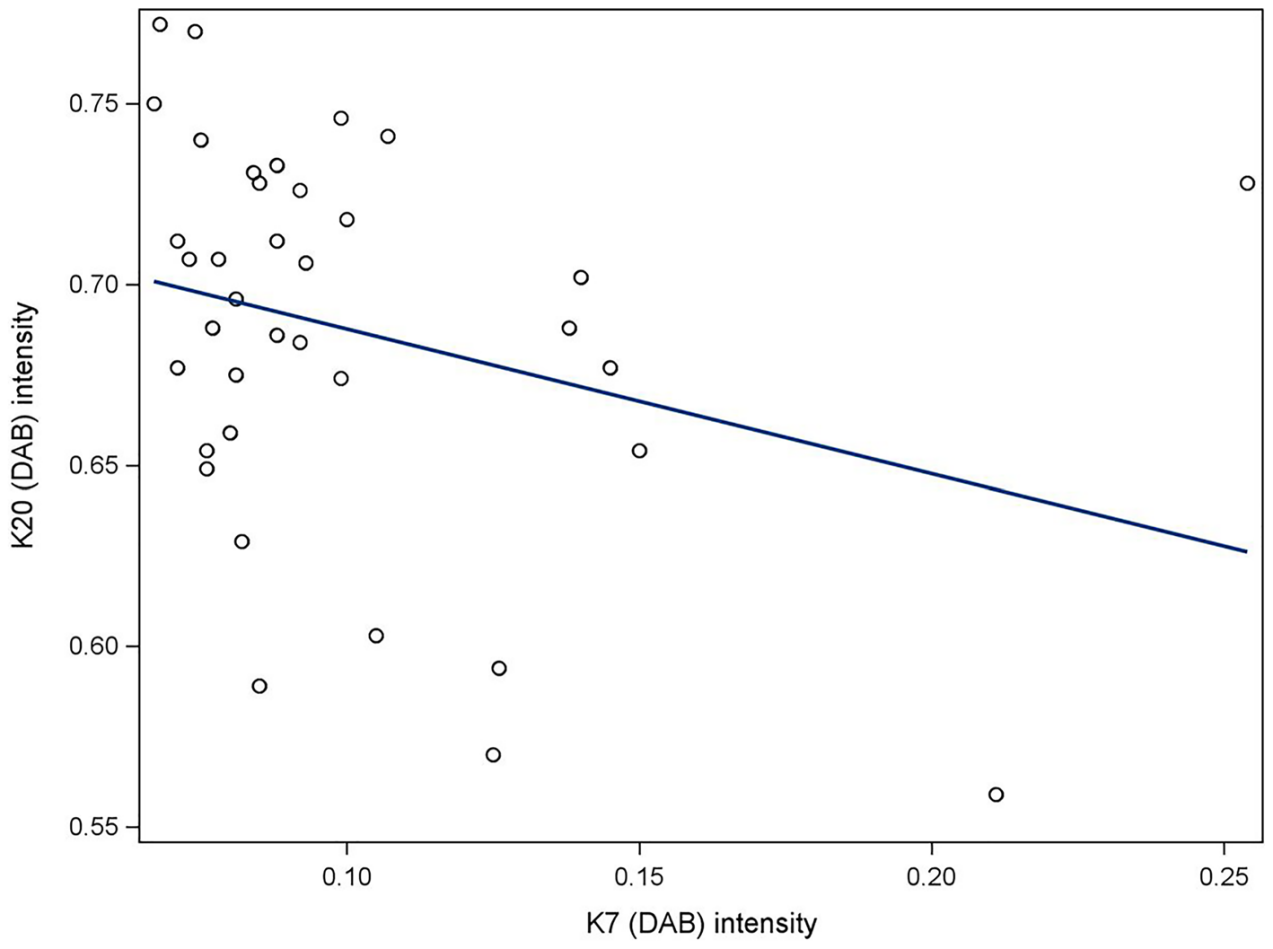
The expressions of K7 and K20 in corresponding annotations of epithelial cells. The linear regression diagram does not suggest a significant correlation between local expression rates of K7 and K20.

The effect of increasing expression of K7 on the expression of K20 was also analyzed. The correlation coefficient between K7 and K20 expressions was negative (− 0.30) but the correlation did not achieve statistical significance (*p* = 0.07) (Fig. 4).

The prognostic value of K7 in the course of IBD was investigated by determining the K7 expression level (percentage) at the time of enrollment and by following the severity of the disease for 3 years. The patients were divided into two groups based on the clinical severity of the disease. Seven patients had mild or moderate disease, and seven patients had severe disease. One patient did not meet the criteria for follow-up because of poor commitment to treatment and follow-up, and the other one had had colectomy before enrollment and only one biopsy of terminal ileum was available.

The association between the clinically estimated severity of the disease and the expression of K7 was calculated in ascending colon, descending colon, and in the colonic segment where the expression was the highest in each patient. As a result, the severity of the disease during the following 3 years was not related to the expression of K7 at the time of enrollment (ascending colon *p* = 0.29, descending colon *p* = 0.59, the colonic segment of the highest K7 expression *p* = 0.62).

## Discussion

In this study we investigated the expression of epithelial intermediate filament protein K7 in defined sites of colon and terminal ileum in patients with IBD. The results showed that the inflammation in bowel wall and the increase in K7 expression correlated significantly in ascending (*p* = 0.004) and descending colon (*p* = 0.0004), but there was no correlation between the inflammatory activity and K7 expression in ileum, transverse colon, or rectum. De novo expression of K7 did not have statistically significant impact on K20 expression in epithelial cells indicating that the colonocyte differentiation state is not pivotal for K7 expression in IBD^41^. This is supported by patchy K7 manifestation lacking the goblet cell specific expression patterns. The prognostic value of K7 in course of IBD was estimated by a 3 year-follow up study and no correlation was observed between K7 expression and the clinical severity of the disease.

We have previously found that K7 expression is associated with IBD^17^. According to the results of the present study, the K7 expression varies based on the anatomical location in intestine being the highest in ascending and descending colon and less in ileum, transverse colon, and rectum where the number of biopsies in this study was probably too small to show the statistically significant difference in expression. To our knowledge, this is the first time when the difference in K7 expression based on the anatomical location in colon is suggested. We have also previously learned that the region of colon has statistically significant effect on standardized uptake value (SUV) in ^18^F-FDG-PET/MRI^34^ implying that glucose uptake in colonic mucosa depends on anatomical location. In addition to the previously mentioned studies^25–27,29^ these findings imply that in pathological states the function of the mucosa is not homogenous throughout the whole colon but the epithelial cells and submucosa act differently in distinct parts of the colon.

Considering a great variation in severity of IBD and response to medication, a personalized treatment would offer a possibility to improve the overall disease outcome and to minimize harmful side-effects of medication. Neutrophil derived fecal calprotectin depicts short-term inflammatory activity in the intestinal mucosa, but there is no reliable prognostic biomarker for IBD^4^. K7 is produced in epithelial cells and thus might reflect the epithelial status better than temporal neutrophil activity. We have previously suggested that high K7 expression in colonic epithelial cells is related to poor response to conservative treatment^17^. However, in the previous study the disease duration of the group that ended up in colectomy varied between 0 and 29 years the median being 6 years for both UC and CD, which is twice the time of follow up in this study. According to an early study^3^, in years 3 to 7 after the IBD diagnosis, ca 20 percent of the patients had active disease every year, the majority of the patients had recurrent relapses and 25 percent were in remission. Even though the only current predictor of the disease course is the previous disease activity^3^, a 3 year follow up may still be too short to see the difference between mild and severe disease, and consequently the monitoring of the patients will be continued. K7 is expressed de novo in inflamed mucosa but the level of expression during and after healing of the mucosa is unknown and should be considered when estimating the potential of K7 as biomarker. In addition, monitoring of changes in K7 expression over the years could produce a profile that might predict the disease outcome. In future, the validation of the value of K7 in the diagnostics and prognosis of IBD requires a prospective study with greater number of patients.

An effective biomarker would be accurate, reproducible, non-invasive, and convenient for sampling^42^. Immunoanalysis for K7 expression is a routine protocol in hospital immunopathology laboratories, but an endoscopy must be performed to obtain the specimens for K7 evaluation. Ileocolonoscopy is required for the assessment of disease activity anyhow, but future studies are warranted to evaluate K7 levels in stool and eligibility of noninvasive keratin assays from fecal matrix.

### Limitations

The major limitation of this study is the small number of patients. In addition, only biopsy specimen with well oriented upright crypts and a sufficiently large number of epithelial cells (> 1000 cells per biopsy) are included. These circumstances may explain why the association between K7 expression and inflammatory activity in transverse colon and rectum fails to reach statistical significance.

## Conclusion

According to this study, the inflammation activity correlates with K7 expression in ascending and descending colon, and on the contrary to colon, there is no K7 expression in inflamed in ileum. The initial K7 level in epithelial specimen does not appear to predict the outcome of IBD during the following 3 years. Long term prospective studies are needed to find out the value of K7 when estimating the prognosis in IBD.

## Supplementary Information

Supplementary Information.

Supplementary Figure 1.

## Data Availability

The datasets generated and analyzed in this study are not publicly available because of the Ethics Committee restrictions but are available from the corresponding author upon reasonable request.
