# Supplementary material for: Keratin 7 expression in different anatomical parts of colonic epithelium in inflammatory bowel diseases and its prognostic value: a 3-year follow-up study
Source: Sci Rep. 2023 Jul 24;13:11979. doi: 10.1038/s41598-023-39066-w (PMC10366087; doi:10.1038/s41598-023-39066-w)
Supplement: Supplementary file 1 — Supplementary Information. [file 41598_2023_39066_MOESM1_ESM.docx]

**Supplementary figure 1**. Keratin 7 distribution in HCT 116 cells. Keratin 7 protein forms a typical filamentous network in the cytoplasm of HCT 116 colon carcinoma cells. HCT-116 cells were grown on microscope cover glasses and fixed in RT PFA for 10 min. Keratin 7 staining was carried out using recombinant anti-cytokeratin 7 antibody EPR17078 (Abcam, Cambridge, UK) followed by Alexa-Fluor 546 IgG 2 secondary antibody (Invitrogen, CA, USA). DNA-staining was carried out by DRAQ5 (Cell Signaling, MA, USA). Samples were analyzed using a Leica TCS SP5 matrix confocal microscope (Leica, Mannheim, Germany).
