# Supplementary figures and images for: Keratin 7 expression in different anatomical parts of colonic epithelium in inflammatory bowel diseases and its prognostic value: a 3-year follow-up study
Source: Sci Rep. 2023 Jul 24;13:11979. doi: 10.1038/s41598-023-39066-w (PMC10366087; doi:10.1038/s41598-023-39066-w)

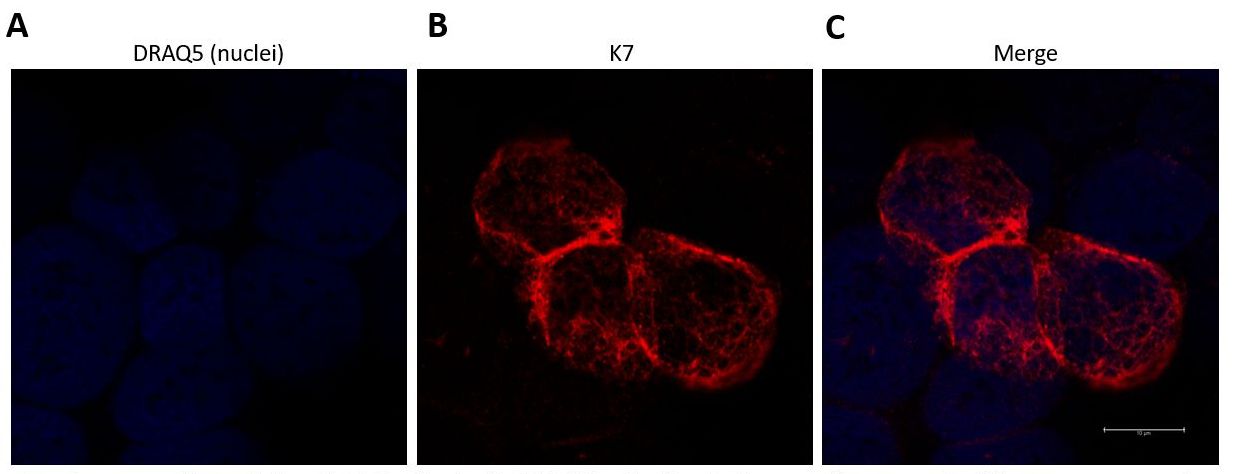

Supplement: Supplementary file 2 — Supplementary Figure 1. [file 41598_2023_39066_MOESM2_ESM.jpg]
